# Comparison of Efficacy of Acupuncture-Related Therapy in the Treatment of Rheumatoid Arthritis: A Network Meta-Analysis of Randomized Controlled Trials

**DOI:** 10.3389/fimmu.2022.829409

**Published:** 2022-03-07

**Authors:** Renhong Wan, Yihua Fan, Anlan Zhao, Yuru Xing, Xiangyuan Huang, Liang Zhou, Ying Wang

**Affiliations:** ^1^ Tianjin University of Traditional Chinese Medicine, Tianjin, China; ^2^ Nanchang Hongdu Hospital of Traditional Chinese Medicine, Nanchang, China

**Keywords:** rheumatoid arthritis, acupuncture-related therapy, DMARDs, randomized controlled trial, network meta-analysis

## Abstract

**Background:**

The refractory, repetitive, and disabling characteristic of rheumatoid arthritis (RA) has seriously influenced the patients’ quality of life, and makes it a major public health problem. As a classic complementary and alternative therapy, acupuncture is usually applied for RA combined with disease-modifying anti-rheumatic drugs (DMARDs). However, there are various types of acupuncture, and the curative effects are different in different acupuncture therapies. In this study, we evaluated the clinical efficacy of different acupuncture therapies combined with DMARDs in the treatment of RA.

**Methods:**

The randomized controlled trials (RCTs) of acupuncture combined with DMARDs in the treatment of RA were searched in both English and Chinese database of PubMed, Cochrane Library, EMBASE, Web of Science, CNKI, VIP database, Wanfang, and SinoMED, up to October 2021. Literature screening, data extraction, and evaluation of the risk of bias were carried out independently by two researchers, and the data were analyzed by Stata14.2 and GeMTC 0.14.3 software.

**Results:**

A total of 32 RCTs were included, including 2,115 RA patients. The results of network meta-analysis were as follows: in terms of improving DAS28 score, Electro-acupuncture + DMARDs has the best efficacy. In terms of improving VAS score, Fire Needle + DMARDs showed the best efficacy. In terms of improving morning stiffness time, acupuncture-related therapies combined with DMARDs were not better than DMARDs alone in improving morning stiffness time in RA patients. In terms of reducing CRP and ESR, Fire Needle + DMARDs showed the best efficacy. In terms of reducing RF, Moxibustion + DMARDs has the best efficacy.

**Conclusions:**

The comprehensive comparison of the outcome indicators in 8 different treatments indicates that electro-acupuncture combined with DMARDs is the best combined therapy in improving DAS28 score, while in terms of improving pain and serological markers, fire needle combined with DMARDs and moxibustion combined with DMARDs were the best combined therapies. However, it is impossible to find out which is better between fire needle and moxibustion due to the limited studies. Clinically, appropriate treatment should be selected according to the actual situation.

**Systematic Review Registration:**

https://www.crd.york.ac.uk/prospero/#recordDetails, CRD42021278233.

## 1 Introduction

Rheumatoid arthritis (RA) is an autoimmune disease characterized by chronic and erosive polyarthritis (1, 2). Its pathological characteristics are inflammatory cell infiltration, joint synovial hyperplasia, and progressive damage in articular cartilage and subchondral bone (3). The aggravation of RA might be accompanied by joint deformities and dysfunction, as well as internal organ injury, such as heart, lungs, and kidneys (4). RA can occur at any age, and mostly between 30 and 60 years old. As a refractory disease, RA seriously affects the quality of life in patients, and it brings a heavy burden to society and economy (5). The prevalence of RA is about 0.3% to 1% (6), and it is closely related to a variety of chronic diseases to bring a huge care burden (7).

At present, there is no radical treatment for RA. The American College of Rheumatology (ACR) recommends disease-modifying anti-rheumatic drugs (DMARDs) against RA (8). However, DMARDs cannot effectively control the progress and relieve clinical symptoms of RA (9). Therefore, how to optimize RA treatment strategies is a major concern for clinicians. Acupuncture is a complementary and alternative therapy based on the meridian theory in traditional Chinese medicine (TCM). It has been widely used in the treatment of knee osteoarthritis (10), ankylosing spondylitis (11, 12), and other arthritis, with a good effect. Acupuncture could improve patient’s symptoms, delay the progression, and reduce pain in the treatment of RA (13, 14). Acupuncture is usually used with the combination of DMARDs for RA. Previous studies have confirmed that acupuncture combined with DMARDs is better than DMARDs (15, 16). However, there are various types of acupuncture, including moxibustion, electro-acupuncture, warm needle, and fire needle acupuncture, and currently, it still lacks a direct comparison of the curative effect in different acupuncture therapies. Therefore, in the real world, which acupuncture therapy should be selected to be combined with DMARDs is still controversial. Network meta-analysis (NMA) is further developed from conventional pairwise meta-analysis (17). According to the current research, NMA could perform direct and indirect comparisons in different acupuncture therapies at the same time, and further comprehensively analyze the results of direct and indirect comparisons to rank the effects of different acupuncture therapies. Therefore, this study used the NMA method to compare the efficacy of different acupuncture therapies in the treatment of RA, to provide evidence for choosing the best combination plans for the clinical treatment of RA.

## 2 Materials and Methods

### 2.1 Registration

This network meta-analysis was conducted according to the Preferred Reporting Items for Systematic Reviews and Meta-Analyses for NMA guidelines (18). The research has been registered in PROSPERO, with the registration website https://www.crd.york.ac.uk/prospero/#recordDetails and registration number CRD42021278233.

### 2.2 Inclusion and Exclusion Criteria

#### 2.2.1 Research Type

Randomized controlled trials (RCTs) in Chinese and English were included.

#### 2.2.2 Research Objects

RA patients conform to clear diagnostic criteria [such as RA diagnostic criteria by the 2010 American College of Rheumatology (ACR) and the European League Against Rheumatism (EULAR) (19)], regardless of gender or age.

#### 2.2.3 Interventions

Patients in the treatment group accepted acupuncture-related therapies combined with DMARDs, including conventional acupuncture, warm needle, electro-acupuncture, fire needle, blood-letting puncture, moxibustion, acupoint embedding, and acupoint injection. Patients in the control group were treated with DMARDs, or DMARDs combined with acupuncture-related therapies. Different DMARDs can be used alone or in combination, but they should be identical in both groups.

#### 2.2.4 Outcome Indicators

The primary outcome indicator was Disease Activity Score of 28 Joints (DAS28) (assessment of RA disease activity by using 28 tender and swollen joint count disease activity score) (20). The secondary outcome indicators were Visual Analogue Scale (VAS), morning stiffness time, serological disease markers including C-reactive protein (CRP), erythrocyte sedimentation rate (ESR), rheumatoid factor (RF), and the occurrence of adverse reactions (AEs).

#### 2.2.5 Exclusion Criteria

- Patients with other rheumatic immune diseases;
- Without clear diagnosis;
- Without outcome indicators;
- With more than two TCM therapies, such as cupping, traditional Chinese herbs, or a combination of two or more acupuncture therapies, such as acupuncture combined with moxibustion and electro-acupuncture combined with moxibustion;
- Repetitively published studies;
- Without complete data in the study even after contacting the authors.

### 2.3 Literature Search Strategy

RCTs of acupuncture combined with DMARDs in the treatment of RA in PubMed, EMBASE, Web of Science, Cochrane Library, China Knowledge Network (CNKI), WanFang, VIP Database, and SinoMed were searched. The search terms were acupuncture, electro-acupuncture, warm needle, fire needle, blood-letting therapy, moxibustion, acupoint catgut embedding, acupoint injection, rheumatoid arthritis, and RA in both Chinese and English. The PubMed database search strategy was shown in **Table 1**.

**Table 1:** PubMed database retrieval strategy.

Number	Search terms
#1	Acupuncture [MeSH]
#2	Acupuncture [Title/Abstract]
#3	Pharmacopuncture [Title/Abstract]
#4	Electro-acupuncture [Title/Abstract]
#5	Warm needle [Title/Abstract]
#6	Fire needle [Title/Abstract]
#7	Blood-letting therapy [Title/Abstract]
#8	Moxibustion [MeSH]
#9	Moxibustion [Title/Abstract]
#10	Auricular application pressure [Title/Abstract]
#11	Auricular needle [Title/Abstract]
#12	Acupoint catgut embedding [Title/Abstract]
#13	Acupoint injection [Title/Abstract]
#14	#1 OR #2 OR #3 OR #4 OR #5 OR #6 OR #7 OR #8 OR #9 OR #10 OR #11 OR #12 OR #13
#15	Rheumatoid arthritis [MeSH]
#16	Rheumatoid arthritis [Title/Abstract]
#17	RA [Title/Abstract]
#18	#15 OR #16 OR #17
#19	#14 AND #18

### 2.4 Literature Screening and Data Extraction

Exclusion of duplicate literature was performed in EndNote X9 software, and then preliminary screening was performed by reading the title and abstract. After that, the full text was further screened to exclude the literature that did not meet the inclusion criteria. For data extraction, two researchers (RW and YF) separately conducted data extraction based on the inclusion and exclusion criteria. If there was any disagreement, the third researcher (LZ) would make a final decision. The data extraction content included title, author, publication year and month, sample size, diagnostic criteria, interventions of treatment group and control group, dosage, course of treatment, and outcome indicators, among others.

### 2.5 Evaluation of the Risk of Bias

The quality evaluation was performed by two separate researchers (RW and YF) using RCT Bias Risk Assessment Tool of the Cochrane System Review Manual Version 5.1.0, and the third researcher (LZ) would assist in judging the divergence between the two researchers. Evaluation items included random sequence generation, allocation concealment, blinding of patients and investigators, blinding of outcome evaluators, incomplete result data, selective reporting, and other biases.

### 2.6 Statistical Analysis

A directly compared meta-analysis was performed using Stata14.2 software. For continuous variables, mean difference (WMD) or standard mean difference (SMD) was used for analysis. *χ* ^2^ test was used to analyze the heterogeneity among the included study results, and *I* ^2^ was used to quantitatively judge the heterogeneity. If *p* ≥ 0.10, *I* ^2^ < 50%, there was no significant heterogeneity between studies, and meta-analysis was performed using a fixed effect model. If *p <* 0.10, *I* ^2^ ≥ 50%, the heterogeneity between studies was considered significant, and a random effect model was used for meta-analysis.

Stata14.2 software was used to make an evidence network diagram to show the comparative relationship in interventions for each outcome indicator. The small sample effects or publication bias was detected by comparison-correction funnel plots. At the same time, network meta-analysis was conducted by GeMTC 0.14.3 software based on the Markov Chain Monte Carlo (MCMC) consistent model under the Bayesian framework. Four chains were used for simulation, with the number of iterations set as 50,000 times (the first 20,000 times for annealing, and the last 30,000 times for sampling), and were estimated and inferred under the assumption that MCMC reached a stable state of convergence evaluated by Potential Scale Reduction Factor (PSRF). The stability and consistency of the results were evaluated using the MCMC inconsistent fitting model.

## 3 Results

### 3.1 Literature Screen Results

**Figure 1:**
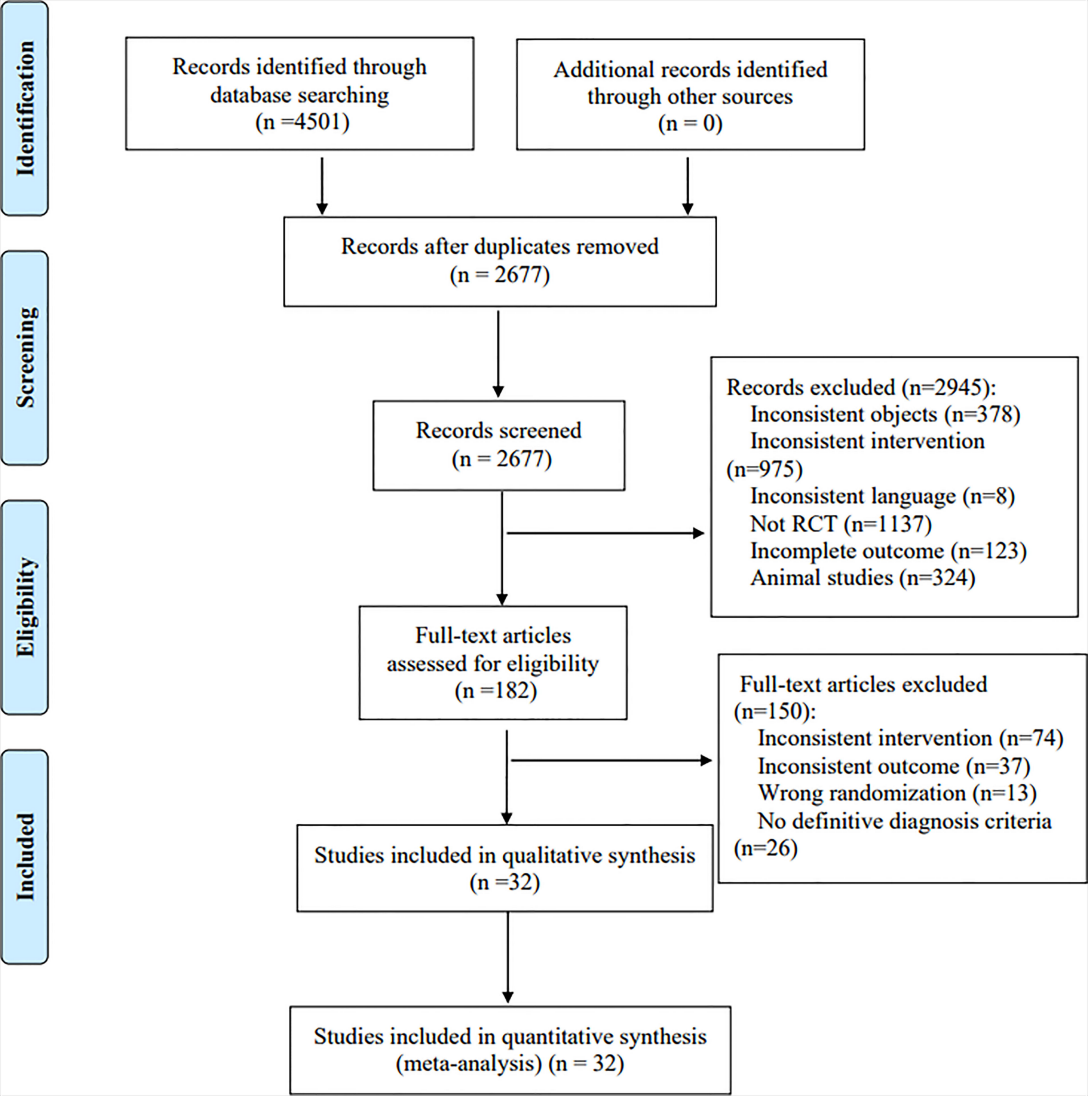
Literature screening process.

A total of 4,501 literatures were retrieved, and 32 RCTs were finally included (21–52) after the preliminary screening and re-screening process, including 2,115 patients. The literature screening process is shown in **Figure 1**.

### 3.2 Characteristics of the Included Studies

In the included 32 studies (21–52), 12 articles reported moxibustion combined with DMARDs, eight articles reported conventional acupuncture combined with DMARDs, five articles reported electro-acupuncture combined with DMARDs, two articles reported acupoint embedding combined with DMARDs, two articles reported fire needle combined with DMARDs, five articles reported warm needle combined with DMARDs, one article reported auricular acupoints combined with DMARDs, and 30 articles reported DMARDs. There is one three-arm study (24) and 31 two-arm studies (21–23, 25–53). Fourteen studies reported DAS28 (21–24, 27, 31, 32, 34, 36–38, 42, 46, 52), 23 studies reported VAS (21–23, 26, 28–30, 32, 33, 35–37, 40–42, 44–50, 52), 14 studies reported morning stiffness (21–24, 26–28, 31, 37, 42, 43, 46, 49, 52), 23 studies reported RF (21–23, 26, 28–30, 32, 33, 35–37, 40–42, 44–50, 52), 27 studies reported CRP (21–24, 27–33, 35, 37, 39–52), 26 studies reported ESR (21–24, 27–33, 35, 37, 39–43, 45–52), and 10 studies reported adverse reactions (23, 28, 30, 32, 34, 35, 39, 43, 50, 51). The characteristics of the included studies are shown in **Table 2**, and the characteristics of intervention measures are shown in **Table 3**.

**Table 2:** Characteristics of the included studies.

Included studies	Diagnostic criteria	Sample (T/C)	Gender (male/female)	Age (years)	Course (years)	Outcome indicators
Ze YY 2020 (21)	ACR	34/32	T: 3/31 C: 4/28	T: 47.29 ± 10.47 C: 48.19 ± 9.87	T: 7.63 ± 7.24 C: 6.23 ± 6.13	①②③④⑤⑥
Gong Y 2019 (22)	ACR/EULAR	20/17	T: 1/19 C: 2/15	T: 46.85 ± 11.3 C: 49.41 ± 10.37	T: 5.44 ± 4.05 C: 6.34 ± 5.11	①②③④⑤⑥
Wang Y 2021 (23)	ACR`	30/31	T: 4/26 C: 5/26	T: 53 ± 8.80 C: 49.39 ± 7.72	T: 10.11 ± 9.02 C: 9.13 ± 9.07	①②③④⑤⑥⑦
Zeng C 2019 (24)	ACR	20/20/20	T1: 5/15 T2: 5/15 C: 6/14	T1: 49 T2: 55 C: 46	T1: 5.3 ± 1.2 T2: 6.0 ± 1.8 C: 5.7 ± 1.4	①②③⑤⑥
Du N 2017 (25)	ACR	32/32	T: 16/16 C: 17/15	T: 52.08 ± 3.82 C: 52.09 ± 3.22	T: 7.42 ± 1.67 C: 7.44 ± 1.63	⑤
Fu HB 2021 (26)	ACR	39/39	T: 8/31 C: 10/29	T: 39.74 ± 4.85 C: 40.04 ± 4.92	T: 3.01 ± 0.35 C: 2.89 ± 0.66	④⑥
Huang S 2013 (27)	ACR	20/20	T: 4/16 C: 7/13	T: 50.36 ± 15.73 C: 49.25 ± 12.54	–	①②③⑤⑥
Jiang L 2020 (28)	ACR	20/20	T: 4/16 C: 2/18	T: 49.35 ± 7.92 C: 51.75 ± 8.43	T: 7.30 ± 4.17 C: 6.15 ± 3.27	①③④⑤⑥⑦
Jing SP 2020 (29)	Practical Arthritis Diagnosis and Therapeutics	45/45	T: 23/22 C: 25/20	T: 51.12 ± 17.86 C: 51.58 ± 17.34		①③④
Li Y 2010 (30)	ACR	40/40	T: 9/31 C: 10/30	T: 36.3 C: 37.7	T: 4.6 C: 4.8	①③④⑦
Lu JG 2021 (31)	ACR	30/30	T: 12/18 C: 14/16	T: 39.25 ± 3.12 C: 39.51 ± 3.15	–	①②③⑥
Lu YL 2016 (32)	ACR	41/39	–	24–62	–	①②③④⑦
Ma ZY 2008 (33)	ACR	40/40	–	30–65	–	①③④
Ma ZY 2016 (34)	ACR	28/29	T: 6/22 C: 5/24	T: 55.6 ± 10.6 C: 54.3 ± 9.8	T: 7.32 ± 1.45 C: 6.53 ± 1.32	②⑦
Mu Y 2020 (35)	ACR/EULAR	28/29	T: 8/20 C: 11/18	T: 54.89 ± 9.43 C: 55.56 ± 11.21	T: 3.71 ± 1.62 C: 3.59 ± 1.01	①③④⑤⑦
Song MX 2017 (36)	ACR	50/50	T: 21/29 C: 19/31	T: 57 ± 11 C: 56 ± 11	T: 4.53 ± 2.30 C: 5.17 ± 2.52	②④
Sun F 2011 (37)	ACR	19/18	T: 6/13 C: 4/14	T: 50.4 ± 9.4 C: 52.3 ± 7.5	T: 0.5 ± 0.3 C: 0.4 ± 0.2	①②③④⑤⑥
Tu JJ 2017 (38)	ACR	30/30	T: 7/23 C: 9/21	T: 53.4 ± 8.9 C: 49.4 ± 11.5	T: 5.36 ± 1.34 C: 6.08 ± 1.65	①②③
Wang SQ 2018 (39)	ACR	41/41	T: 15/26 C: 16/25	T: 48.5 ± 1.3 C: 48.6 ± 1.5	T: 8.2 ± 1.2 C: 8.2 ± 1.4	①③⑦
Wang GQ 2017 (40)	ACR	54/54	T: 24/30 C: 21/33	T: 43.8 ± 5.6 C: 43.8 ± 5.7	T: 5.7 ± 2.1 C: 5.6 ± 2.0	①③④
Wang JJ 2021 (41)	ACR	46/46	T: 30/16 C: 28/18	T: 47.93 ± 1.82 C: 48.26 ± 1.73	T: 6.95 ± 1.07 C: 7.19 ± 1.15	①③④
Wang YY 2020 (42)	ACR	33/31	T: 5/28 C: 5/26	T: 52.64 ± 8.859 C: 51.13 ± 9.029	T: 10.15 ± 8.97 C: 8.89 ± 8.841	①②③④⑤⑥
Wu Y 2016 (43)	ACR	53/53	T: 14/39 C: 15/38	T: 39.9 C: 41.1	T: 8.3 C: 7.9	①③⑥⑦
Xiao J 2019 (44)	ACR	40/40	T: 13/27 C: 9/31	T: 39.2 ± 18.3 C: 38.6 ± 16.5	–	①④⑤
Xiong Y 2019 (45)	ACR	16/16	T: 2/14 C: 3/13	T: 52.4 ± 13.1 C: 49.8 ± 14	T: 5.25 ± 2 C: 5.31 ± 2	①③④
Yang CH 2016 (46)	ACR	16/16	T: 2/14 C: 3/13	T: 52.4 ± 13.1 C: 49.8 ± 14	T: 5.25 ± 2 C: 5.31 ± 2	①②③④⑤⑥
Zang XL 2016 (47)	ACR	41/41	T: 17/24 C: 16/25	T: 42.7 ± 3.2 C: 42.6 ± 2.4	–	①③④
Zhang M 2021 (48)	ACR	30/30	T: 5/25 C: 6/24	T: 50 ± 13 C: 54 ± 11	T: 11.2 ± 7.2 C: 10.5 ± 6.0	①③④
Zhang YT 2019 (49)	ACR	60/60	T: 24/36 C: 25/35	T: 58.32 ± 5.24 C: 57.41 ± 5.46	T: 4.31 ± 1.58 C: 4.24 ± 1.54	①③④⑥
Zhang YD 2019 (50)	Guidelines for the diagnosis and treatment of rheumatoid arthritis	36/36	T: 15/21 C: 14/22	T: 49.39 ± 5.26 C: 48.97 ± 5.26	T: 10.29 ± 1.36 C: 10.1 ± 1.58	①③④
Zheng HY 2017 (51)	ACR	28/27	T: 5/23 C: 6/21	T: 45.77 ± 6.83 C: 47.32 ± 6.25	T: 8.71 ± 2.81 C: 8.19 ± 2.67	①③⑦
Zhou MQ 2019 (52)	ACR	20/20	T: 8/12 C: 6/14	T: 49.85 ± 11.997 C: 56.10 ± 8.522	T: 54.95 ± 52.52 C: 90.6 ± 61.86	① ②③④⑤⑥

T, Treatment Group; C, Control Group; ① CRP; ② DAS28; ③ ESR; ④ RF; ⑤ VAS; ⑥ Morning stiffness; ⑦ AEs.

**Table 3:** Characteristics of interventions of included studies.

Included studies	Design	Interventions		Treatment course (week)
		T	C	
Ze YY 2020 (21)	Two-arm	Moxibustion (ST36, BL23, Ashi points, twice/week)	Methotrexate 7.5 mg, once a week; Leflunomide 10 mg, once day	8
Gong Y 2019 (22)	Two-arm	Moxibustion (ST36, BL23, Ashi points, twice/week)	Methotrexate (2.5 mg/pill) or leflunomide (10 mg/pill)	8
Wang Y 2021 (23)	Two-arm	Moxibustion (ST36, BL23, Ashi points, twice/week)	Methotrexate 7.5 mg, once a week; Leflunomide 10 mg, once day	8
Zeng C 2019 (24)	Three-arm	Acupuncture/electro-acupuncture (DU14, BL23, ST36, DU4, BL20, once every 2 days, 30 min once)	Methotrexate 7.5 mg, once a week; Leflunomide 20 mg, once day	12
Du N 2017 (25)	Two-arm	Electro-acupuncture (BL18, BL23, GB39, ST36, LI4, three times a week, 20 min once)	Leflunomide 10 mg, once day	12
Fu HB 2021 (26)	Two-arm	Warm needle (RN4, BL18, BL20, BL23, ST36, once a day)	Methotrexate 7.5 mg, once a week; Leflunomide 10 mg, once day	4
Huang S 2013 (27)	Two-arm	Moxibustion (RN4, ST36, five times a week, 30 min once)	Leflunomide 20 mg, once day	12
Jiang L 2020 (28)	Two-arm	Acupuncture (ST36, SP9, SP8, SP 10, three times a week, 30 min once)	Methotrexate 10 mg, once a week	12
Jing SP 2020 (29)	Two-arm	Acupuncture (ST36, DU14, LI4, GB34, SP10, once a day, 30 min once)	Methotrexate 10 mg, once a week	8
Li Y 2010 (30)	Two-arm	Acupuncture (ST36, BL23, DU14, SP10, LI4 five times a week, 30 min once)	Leflunomide 20 mg, once day	4
Lu JG 2021 (31)	Two-arm	Auricular needle (adrenal gland, endocrine, small occipital nerve point, subcortex of cardiovascular system, pressing 4 times a day, 20 presses each time)	Methotrexate 10 mg, once a week	12
Lu YL 2016 (32)	Two-arm	Warm needle (BL18, BL20, BL23, ST36, Ashi points, once a day, 30 min once)	Methotrexate 10 mg, once a week	4
Ma ZY 2008 (33)	Two-arm	Electro-acupuncture (BL18, BL20, BL23, ST36, LI4, five times a week, 30 min once)	Methotrexate 10 mg, once a week	4
Ma ZY 2016 (34)	Two-arm	Acupoint catgut embedding (ST36, BL23, once every 15 days)	Leflunomide 20 mg, once day	12
Mu Y 2020 (35)	Two-arm	Fire needle (Ashi points in three Hand-Yang meridians, twice a week, 30 min once)	Methotrexate 7.5 mg, once a week; Leflunomide 20 mg, once day; acupuncture (SJ5, SJ4, LI5, SI4, BL23, RN4, twice a week, 30 min once)	12
Song MX 2017 (36)	Two-arm	Warm needle (ST36, LI11, SJ5, Xiyan, BL60, once every 2 days)	Methotrexate 7.5 mg, once a week	2
Sun F 2011 (37)	Two-arm	Moxibustion (RN4, ST36, five times a week, 30 min once)	Methotrexate 10 mg, once a week	12
Tu JJ 2017 (38)	Two-arm	Acupoint catgut embedding (ST36, RN4, BL23, once every 15 days)	Leflunomide 20 mg, once day	12
Wang SQ 2018 (39)	Two-arm	Acupuncture (EX-UE9, once every 2 days, 30 min once)	Leflunomide 10 mg, five times a day for the first 3 days, and once or twice after that	8
Wang GQ 2017 (40)	Two-arm	Moxibustion (BL20, BL23, RN4, ST36, Ashi points, once a day, 30 min once)	Methotrexate 10 mg, once a week	4-8
Wang JJ 2021 (41)	Two-arm	Moxibustion (RN4, ST36, five times a week, 30 min once)	Methotrexate 7.5 mg, once a week; Leflunomide 20 mg, once day	4
Wang YY 2020 (42)	Two-arm	Moxibustion (RN4, ST36, Ashi points, twice a week, 30 min once)	Methotrexate 7.5 mg, once a week; Leflunomide 10 mg, once day	8
Wu Y 2016 (43)	Two-arm	Warm needle (ST36, DU14, SP6, GB20, Ashi points, once every 2 days, 30 min once)	Methotrexate 10 mg, once a week	16
Xiao J 2019 (44)	Two-arm	Warm needle (BL23, ST36, GB34, Ashi points, twice a week, 30 min once)	Methotrexate 10 mg, once a week	24
Xiong Y 2019 (45)	Two-arm	Moxibustion (BL23, ST36, Ashi points, twice a week, 30 min once)	Methotrexate 10 mg, once a week; Leflunomide 10 mg, once day	12
Yang CH 2016 (46)	Two-arm	Moxibustion (ST36, BL23, BL43, BL13, Ashi points, twice a week)	Methotrexate 7.5 mg, once a week; or Leflunomide 10 mg, once day	12
Zang XL 2016 (47)	Two-arm	Electro-acupuncture (ST36, KI3, BL18, BL20, Ashi points, 30 min once)	Methotrexate 10 mg, once a week	12
Zhang M 2021 (48)	Two-arm	Moxibustion (BL23, ST36, SP6, Ashi points, three times a week, 30 min once)	Leflunomide 20 mg, once day	8
Zhang YT 2019 (49)	Two-arm	Acupuncture (BL18, BL20, BL23, RN4, ST36, once a day, 30 min once)	Methotrexate 7.5 mg, once a week; Leflunomide 10 mg, once day	4
Zhang YD 2019 (50)	Two-arm	Fire needle (Ex-B5, Ashi points)	Leflunomide 20 mg, once day; acupuncture (Ex-B5, Ashi points)	4
Zheng HY 2017 (51)	Two-arm	Electro-acupuncture (EX-UE9, once every 2 days, 30 min once)	Leflunomide 10 mg, once day	6
Zhou MQ 2019 (52)	Two-arm	Moxibustion (BL23, ST36, Ashi points, twice a week)	Methotrexate 7.5 mg, once a week; or Leflunomide 10 mg, once day	12

T, Treatment Group; C, Control Group.

### 3.3 Results of the Risk of Bias

**Figure 2:**
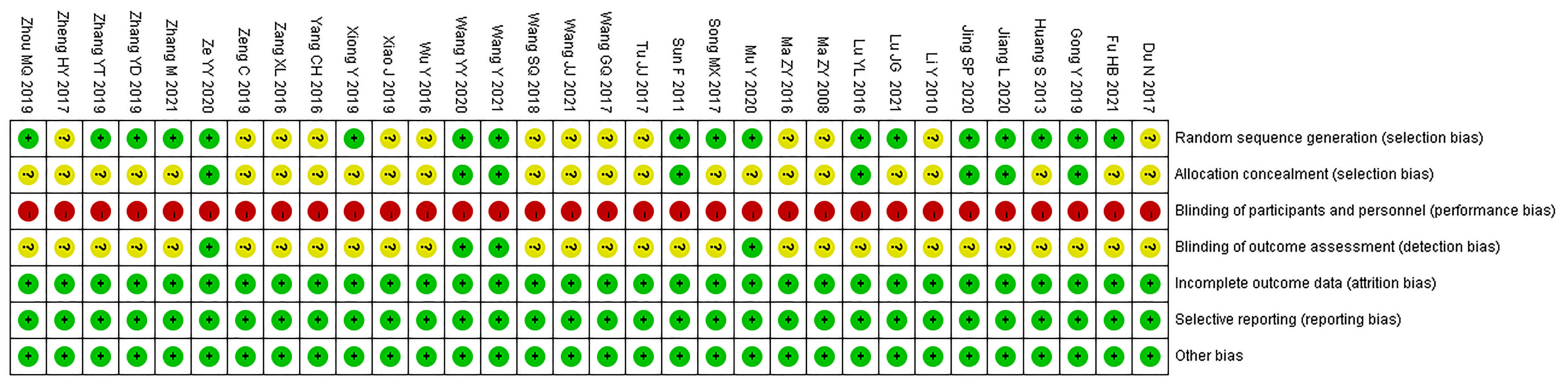
Results of the risk of bias evaluation.

For random sequence generation, random number tables were used in 12 studies (22, 26–28, 31, 32, 35, 36, 45, 49, 50, 52), random numbers were generated by computer in 6 studies (21, 23, 29, 37, 42, 48), and the remaining 14 studies (24, 25, 30, 33, 34, 38–41, 43, 44, 46, 47, 51) only mentioned the word “random”. For allocation concealment, six studies (21–23, 28, 37, 42) used sealed opaque envelopes, one study (32) used central allocation, one study (29) used lottery, and the remaining 24 studies did not report allocation concealment. For blinding of investigators and participants, due to the limitation of interventions, double-blind was not applied in all studies. For blinding of the outcome assessors, the outcome assessors were blinded in four studies (21, 23, 35, 42), and the remaining 28 studies did not report blinding of the outcome evaluators. For incomplete reporting, selective reporting, and other biases, all the 32 studies (21–52) reported complete data, without selective reporting and others bias. The results of the risk of bias evaluation are shown in **Figure 2**.

### 3.4 Directly Compared Meta-Analysis Results

#### 3.4.1 DSA28 Scores

The results of meta-analysis showed that the DSA28 scores of the Moxibustion + DMARDs group and Acupuncture + DMARDs group were lower than that of the DMARDs group (*p* < 0.05). The DSA28 scores of the Warm Needle + DMARDs group and Acupoint catgut embedding + DMARDs group had no difference compared with that of the DMARDs group (*p* > 0.05). Descriptive analysis results showed that the DSA28 scores of the Electro-acupuncture + DMARDs group and Auricular Needle + DMARDs group were lower than that of the DMARDs group (*p* < 0.05). The DSA28 scores of the Electro-acupuncture + DMARDs group were lower than that of the Acupuncture + DMARDs group (*p* < 0.05). See supplementary materials (**Table S2**).

#### 3.4.2 VAS Scores

The results of meta-analysis showed that the VAS scores of the Moxibustion + DMARDs group, Acupuncture + DMARDs group, and Electro-acupuncture + DMARDs group were lower than that of the DMARDs group (*p* < 0.05). Descriptive analysis results showed that the VAS scores of the Warm Needle + DMARDs group and the Fire Needle + DMARDs group were lower than that of the DMARDs group (*p* < 0.05). See supplementary materials (**Table S2**).

#### 3.4.3 Morning Stiffness Time

The results of meta-analysis showed that morning stiffness time in the Acupuncture + DMARDs group and Warm Needle + DMARDs group was lower than that of the DMARDs group (*p* < 0.05). The morning stiffness time in the Moxibustion + DMARDs group was not different from that of the DMARDs group (*p* > 0.05). Descriptive analysis results showed that the morning stiffness time of the Electro-acupuncture + DMARDs group and Auricular needle + DMARDs group was lower than that of the DMARDs group (*p* < 0.05). The morning stiffness time of the Electro-acupuncture + DMARDs group was lower than that of the Acupuncture + DMARDs group (*p* < 0.05). See supplementary materials (**Table S2**).

#### 3.4.4 CRP

The results of meta-analysis showed that the CRP of the Moxibustion + DMARDs group, Acupuncture + DMARDs group, Electro-acupuncture + DMARDs group, and Warm Needle + DMARDs group were lower than that of the DMARDs group. The CRP of the Fire Needle + DMARDs group was lower than that of the Acupuncture + DMARDs group (*p* < 0.05). Descriptive analysis results showed that the CRP of the Auricular needle + DMARDs group was lower than that of the DMARDs group. The CRP of the Electro-acupuncture + DMARDs group was lower than that of the Acupuncture + DMARDs group (*p* < 0.05). See supplementary materials (**Table S2**).

#### 3.4.5 ESR

The results of meta-analysis showed that the ESR of the Moxibustion + DMARDs group, Acupuncture + DMARDs group, Electro-acupuncture + DMARDs group, and Warm Needle + DMARDs group was lower than that of the DMARDs group. The ESR of the Fire Needle + DMARDs group was lower than that of the Acupuncture + DMARDs group (*p* < 0.05). Descriptive analysis results showed that the ESR of the Auricular needle + DMARDs group was lower than that of the DMARDs group. The ESR of the Electro-acupuncture + DMARDs group was lower than that of the Acupuncture + DMARDs group (*p* < 0.05). See supplementary materials (**Table S2**).

#### 3.4.6 RF

The results of meta-analysis showed that the RF of the Moxibustion + DMARDs group, Acupuncture + DMARDs group, Electro-acupuncture + DMARDs group, and Warm Needle + DMARDs group was lower than that of the DMARDs group (*p* < 0.05). There was no difference in RF in the Fire needle + DMARDs group as compared to the Acupuncture + DMARDs group (*p* > 0.05). See supplementary materials (**Table S2**).

#### 3.4.7 Subgroup Analysis

To further explore the effect of different treatment duration on the results, we conducted subgroup analysis. Due to the small number of studies involved in some intervention schemes, subgroup analysis of all outcome indicators was not possible, so we only conducted a subgroup analysis of the outcome indicators involved in the Moxibustion + DMARDs group vs. DMARDs group. The results showed that for DAS28 scores, CRP, ESR, and RF, the Moxibustion + DMARDs group was superior to the DMARDs group regardless of the treatment duration, and the long treatment course (12 week) was superior to the short treatment course (≥4 weeks and ≤8 weeks) (*p* < 0.05). For VAS scores and morning stiffness time, the Moxibustion + DMARDs group was lower than the DMARDs group in the short course of treatment (4 weeks). However, there was no difference between the Moxibustion + DMARDs group and the DMARDs group in the long course of treatment (18 weeks) (*p* > 0.05). See supplementary materials (**Table S3**).

#### 3.4.8 Heterogeneity Analysis

In the directly compared meta-analysis, some results were heterogeneous. Through the analysis of the original data, it was found that there may be methodological heterogeneity due to less description of the blind method and allocation concealment in the included studies. At the same time, the clinical heterogeneity may be caused by factors such as the inclusion population, acupoints, and operation methods. However, due to the lack of specific description of these details in the original study and the small number of studies in some results, it was impossible to further explore the source of heterogeneity by subgroup analysis. Although we have carried out subgroup analysis on some results, all heterogeneity has not been eliminated. However, we did a sensitivity analysis and found that the results were stable after we excluded either study. Therefore, we can ignore this heterogeneity and adopt a random effect model for meta-analysis.

### 3.5 Results of Network Meta-Analysis

#### 3.5.1 Evidence Network Diagram

**Figure 3:**
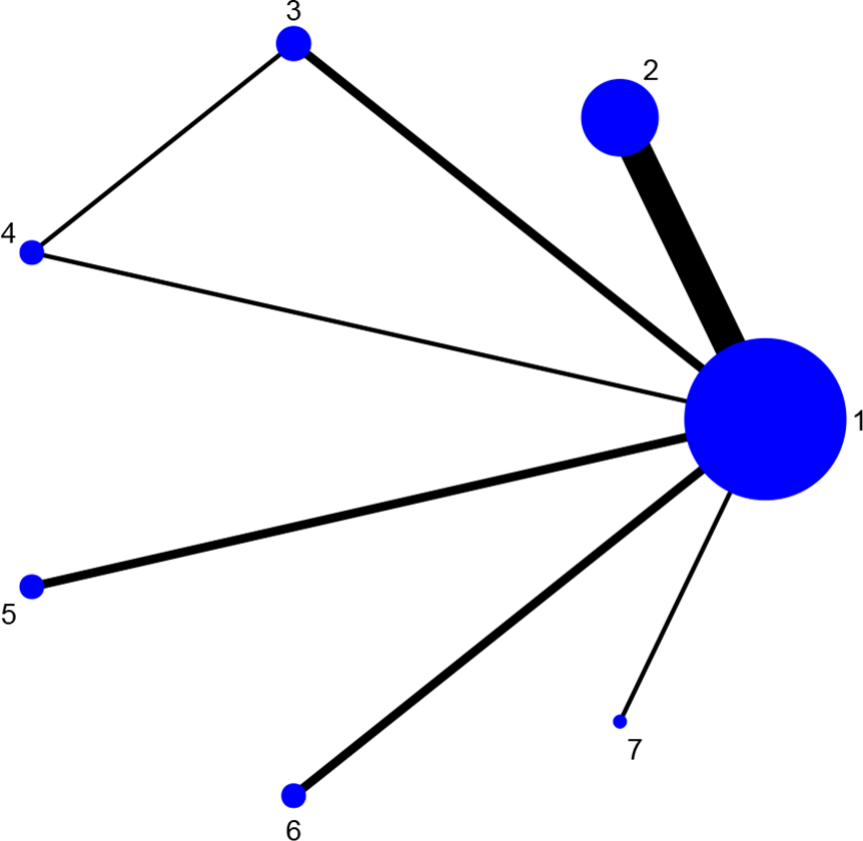
Evidence network diagram of DAS28 of different acupuncture therapies against RA. 1, DMARDs; 2, Moxibustion + DMARDs; 3, Acupuncture + DMARDs; 4, Electro-acupuncture + DMARDs; 5, Warm needle + DMARDs; 6, Acupoint catgut embedding + DMARDs; 7, Auricular needle + DMARDs.

**Figure 4:**
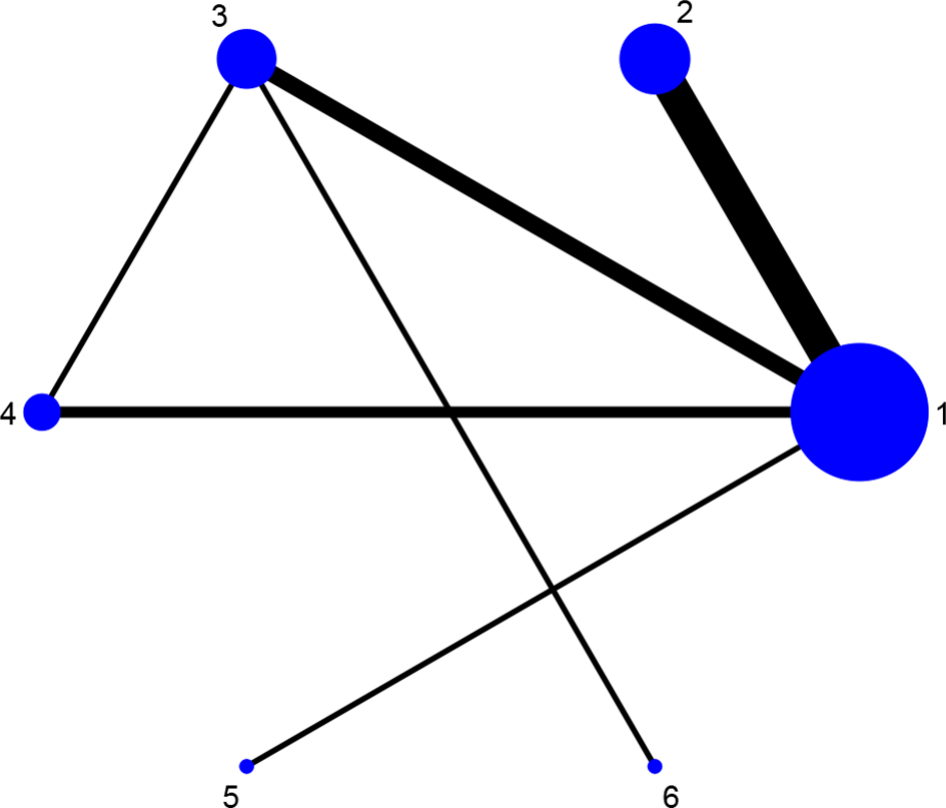
Evidence network diagram of VAS of different acupuncture therapies against RA. 1, DMARDs; 2, Moxibustion + DMARDs; 3, Acupuncture + DMARDs; 4, Electro-acupuncture + DMARDs; 5, Warm needle + DMARDs; 6, Fire needle + DMARDs.

**Figure 5:**
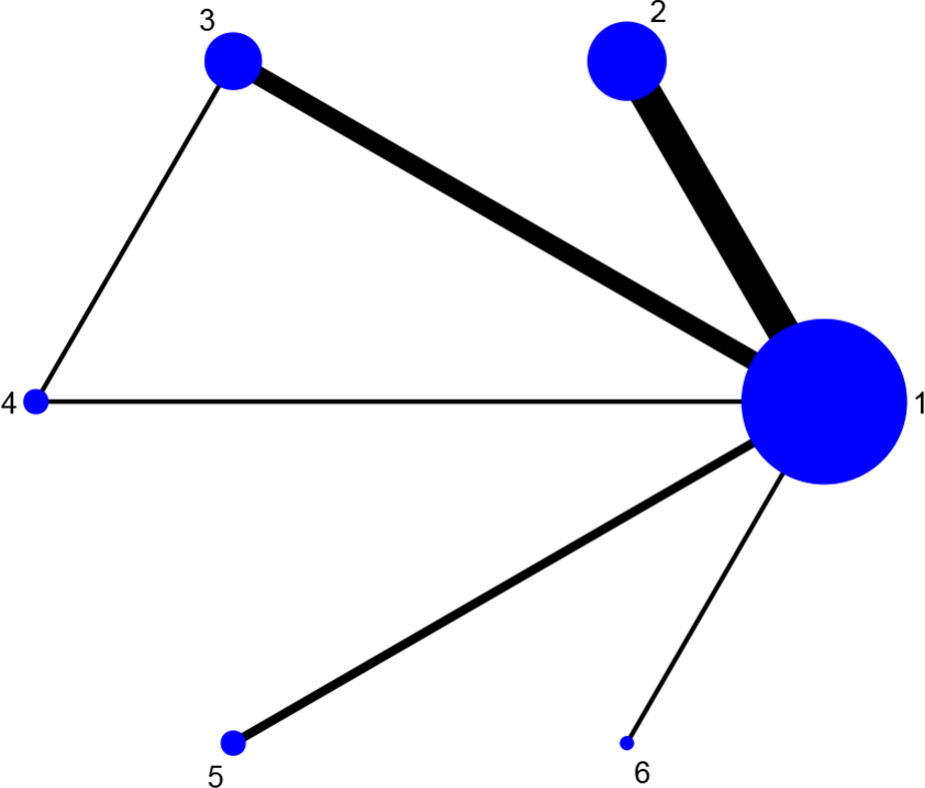
Evidence network diagram of morning stiffness time of different acupuncture therapies against RA. 1, DMARDs; 2, Moxibustion + DMARDs; 3, Acupuncture + DMARDs; 4, Electro-acupuncture + DMARDs; 5, Warm needle + DMARDs; 6, Auricular needle + DMARDs.

**Figure 6:**
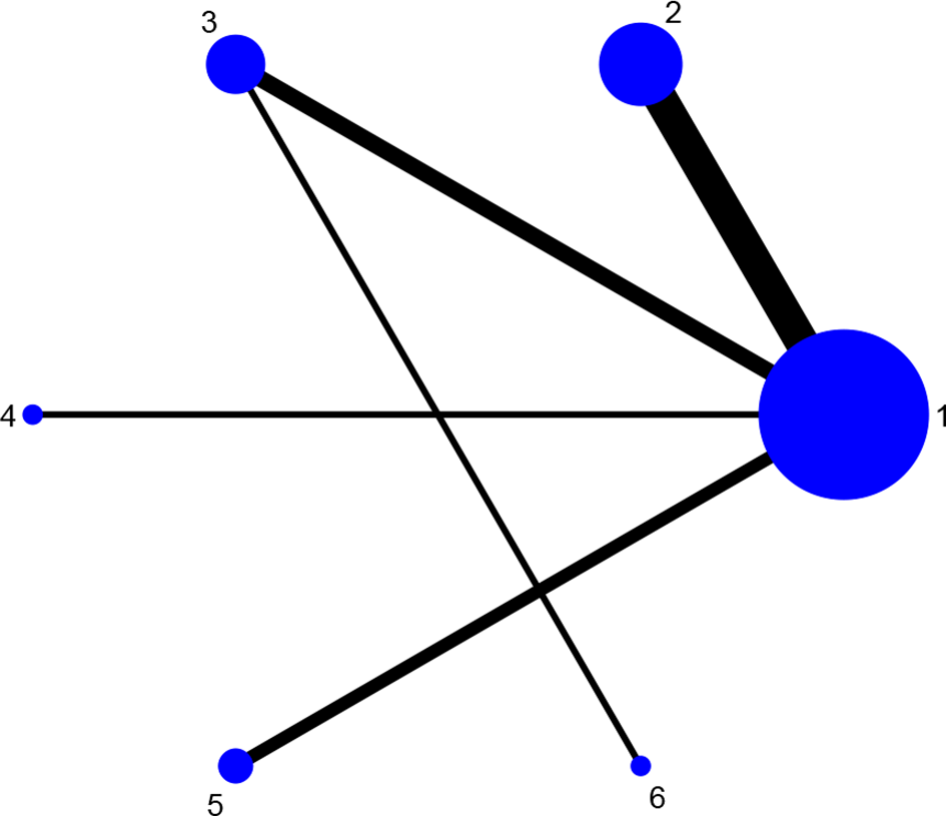
Evidence network diagram of RF of different acupuncture therapies against RA. 1, DMARDs; 2, Moxibustion + DMARDs; 3, Acupuncture + DMARDs; 4, Electro-acupuncture + DMARDs; 5, Warm needle + DMARDs; 6, Fire needle + DMARDs.

**Figure 7:**
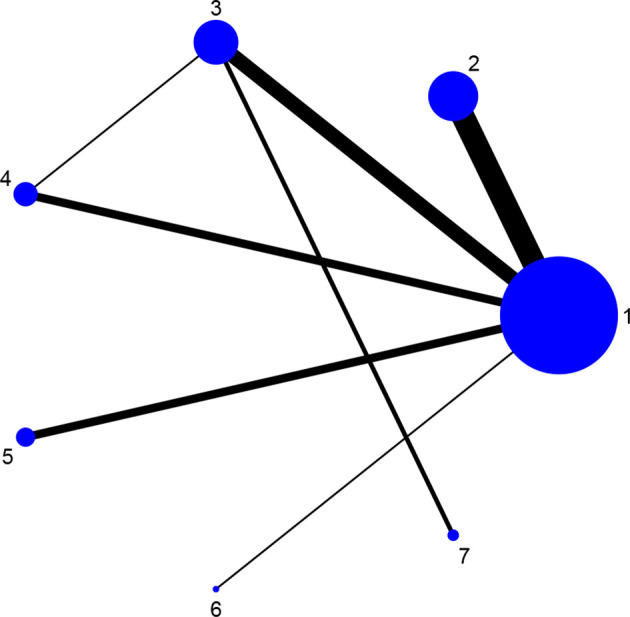
Evidence network diagram of CRP of different acupuncture therapies against RA. 1, DMARDs; 2, Moxibustion + DMARDs; 3, Acupuncture + DMARDs; 4, Electro-acupuncture + DMARDs; 5, Warm needle + DMARDs; 6, Auricular needle + DMARDs; 7, Fire needle + DMARDs.

**Figure 8:**
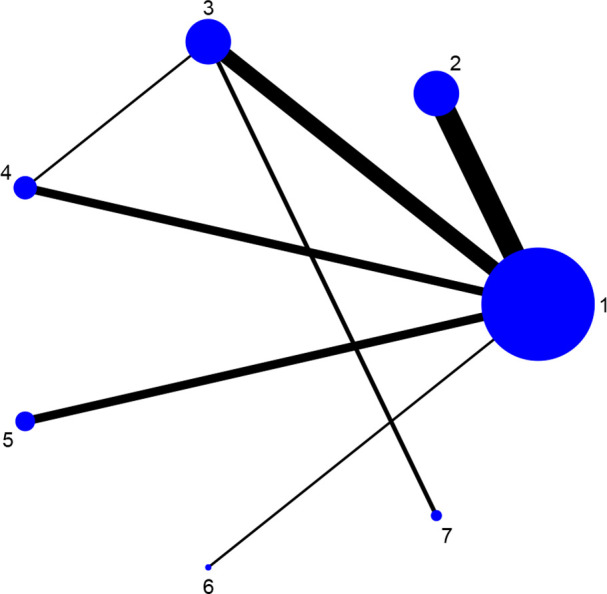
Evidence network diagram of ESR of different acupuncture therapies against RA. 1, DMARDs; 2, Moxibustion + DMARDs; 3, Acupuncture + DMARDs; 4, Electro-acupuncture + DMARDs; 5, Warm needle + DMARDs; 6, Auricular needle + DMARDs; 7, Fire needle + DMARDs.

Fourteen studies reported DAS28 (21–24, 27, 31, 32, 34, 36–38, 42, 46, 52), involving 7 therapies, to form a closed loop, namely, DMARDs and Acupuncture + DMARDs and Electro-acupuncture + DMARDs. Twenty-three studies reported VAS (21–23, 26, 28–30, 32, 33, 35–37, 40–42, 44–50, 52), involving 6 therapies, to form a closed loop, namely, DMARDs and Acupuncture + DMARDs and Electro-acupuncture + DMARDs. Fourteen studies reported the time of morning stiffness (21–24, 26–28, 31, 37, 42, 43, 46, 49, 52), involving 6 treatment options, and they formed a closed loop, namely, DMARDs and Acupuncture + DMARDs and Electro-acupuncture + DMARDs. Twenty-three studies reported RF (21–23, 26, 28–30, 32, 33, 35–37, 40–42, 44–50, 52), involving 6 treatments, and failed to form a closed loop. Twenty-eight studies reported CRP (21–24, 27–33, 35, 37–52), involving 7 treatment options, to form a closed loop, namely, DMARDs and Acupuncture + DMARDs and Electro-acupuncture + DMARDs. Twenty-seven studies reported ESR (21–24, 27–33, 35, 37–43, 45–52), involving 7 treatment options, and formed a closed loop, namely, DMARDs and Acupuncture + DMARDs and Electro-acupuncture + DMARDs. The thicker the line between the two interventions was, the greater the number of studies between the two measures was. The larger the node was, the larger the research sample size was. Except for DMARDs, Acupuncture + DMARDs, and Electro-acupuncture + DMARDs, there was no closed loop among the other interventions, which indicated that there was no direct comparison between those interventions, as shown in **Figures 3**–**8**.

#### 3.5.2 Results of DSA28 Scores

Fourteen studies reported DAS28 (21–24, 27, 31, 32, 34, 36–38, 42, 46, 52). The convergence evaluation results showed that the PSRF value was close to 1, and the inconsistent fit model result was similar to the consistent fit model (*p* = 0.407 > 0.05), indicating fine stability and consistency of results, so the MCMC fitting consistency model was used for analysis. The results showed that Moxibustion + DMARDs was better than DMARDs, and Electro-acupuncture + DMARDs was better than Moxibustion + DMARDs, DMARDs, and Acupoint catgut embedding + DMARDs. There was no statistically significant difference between the other therapies, as seen in **Table 4**. The probability ranking results of improving DSA28 score were as follows: Electro-acupuncture + DMARDs > Auricular needle + DMARDs > Acupuncture + DMARDs > Moxibustion + DMARDs > Warm needle + DMARDs > DMARDs > Acupoint catgut embedding + DMARDs, as shown in **Table 5**.

**Table 4:** NMA results of DSA28 scores.

A	−0.62 (−1.17, −0.10)	−0.92 (−1.97, 0.08)	−2.16 (−3.51, −0.87)	−0.79 (−1.75, 0.14)	0.29 (−0.63, 1.18)	−1.22 (−2.49, 0.06)
**0.62 (0.10, 1.17)**	B	−0.30 (−1.46, 0.83)	−1.54 (−2.98, −0.12)	−0.17 (−1.28, 0.92)	0.92 (−0.16, 1.97)	−0.59 (−1.97, 0.80)
0.92 (−0.08, 1.97)	0.30 (−0.83, 1.46)	C	−1.25 (−2.55, 0.07)	0.14 (−1.28, 1.50)	1.22 (−0.12, 2.59)	−0.29 (−1.88, 1.36)
**2.16 (0.87, 3.51)**	**1.54 (0.12, 2.98)**	1.25 (−0.07, 2.55)	D	1.37 (−0.24, 3.01)	2.45 (0.88, 4.10)	0.96 (−0.89, 2.80)
0.79 (−0.14, 1.75)	0.17 (−0.92, 1.28)	−0.14 (−1.50, 1.28)	−1.37 (−3.01, 0.24)	E	1.09 (−0.22, 2.42)	−0.43 (−2.03, 1.18)
−0.29 (−1.18, 0.63)	−0.92 (−1.97, 0.16)	−1.22 (−2.59, 0.12)	**−2.45 (−4.10, −0.88)**	−1.09 (−2.42, 0.22)	F	−1.52 (−3.08, 0.09)
1.22 (−0.06, 2.49)	0.59 (−0.80, 1.97)	0.29 (−1.36, 1.88)	−0.96 (−2.80, 0.89)	0.43 (−1.18, 2.03)	1.52 (−0.09, 3.08)	G

The above data represent the confidence interval. The bold font indicates that there was a statistically significant difference between the two treatments. A, DMARDs, B, Moxibustion + DMARDs, C, Acupuncture + DMARDs, D, Electro-acupuncture + DMARDs, E, Warm needle + DMARDs, F, Acupoint catgut embedding + DMARDs, G, Auricular needle + DMARDs.

**Table 5:** Probability ranking results of DSA28 scores.

Drug	Rank 1	Rank 2	Rank 3	Rank 4	Rank 5	Rank 6	Rank 7
A	0.2	**0.72**	0.07	0.01	0	0	0
B	0	0.03	0.41	**0.35**	0.17	0.04	0
C	0.01	0.03	0.16	0.22	**0.33**	0.23	0.01
D	0	0	0.01	0.01	0.03	0.12	**0.84**
E	0.02	0.04	**0.23**	0.28	0.26	0.15	0.03
F	**0.75**	0.16	0.05	0.02	0.01	0	0
G	0.01	0.02	0.07	0.12	0.21	**0.45**	0.12

The bold font represents the probability ranking of the therapies. A, DMARDs, B, Moxibustion + DMARDs, C, Acupuncture + DMARDs, D, Electro-acupuncture + DMARDs, E, Warm needle + DMARDs, F, Acupoint catgut embedding + DMARDs, G, Auricular needle + DMARDs.

#### 3.5.3 Results of VAS Scores

Twenty-three studies reported VAS (21–23, 26, 28–30, 32, 33, 35–37, 40–42, 44–50, 52). The results of convergence evaluation showed that the PSRF value was close to 1, and the inconsistent fit model result was similar to the consistent fit model (*p* = 0.592 > 0.05), indicating fine stability and consistency of results, so the MCMC fitting consistency model was used for analysis. The results showed that Moxibustion + DMARDs was better than DMARDs, Acupuncture + DMARDs was better than DMARDs, Electro-acupuncture + DMARDs was better than DMARDs, and Fire needle + DMARDs was better than DMARDs, Moxibustion + DMARDs, and Acupuncture + DMARDs. There was no statistically significant difference between the other therapies, as seen in **Table 6**. The probability ranking results of improving VAS scores were as follows: Fire needle + DMARDs > Electro-acupuncture + DMARDs > Acupuncture + DMARDs > Moxibustion + DMARDs > Warm needle + DMARDs > DMARDs, as shown in **Table 7**.

**Table 6:** NMA results of VAS scores.

A	−1.01 (−1.82, −0.19)	−1.64 (−2.83, −0.38)	−2.18 (−3.47, −0.66)	−1.24 (−3.19, 0.71)	−3.58 (−5.81, −1.31)
**1.01 (0.19, 1.82)**	B	−0.62 (−2.06, 0.87)	−1.16 (−2.67, 0.58)	−0.22 (−2.37, 1.87)	−2.58 (−4.90, −0.17)
**1.64 (0.38, 2.83)**	0.62 (−0.87, 2.06)	C	−0.54 (−2.10, 1.08)	0.42 (−1.92, 2.71)	−1.94 (−3.79, −0.07)
**2.18 (0.66, 3.47)**	1.16 (−0.58, 2.67)	0.54 (−1.08, 2.10)	D	0.94 (−1.57, 3.21)	−1.41 (−3.93, 1.07)
1.24 (−0.71, 3.19)	0.22 (−1.87, 2.37)	−0.42 (−2.71, 1.92)	−0.94 (−3.21, 1.57)	E	−2.37 (−5.23, 0.63)
**3.58 (1.31, 5.81)**	**2.58 (0.17, 4.90)**	**1.94 (0.07, 3.79)**	1.41 (−1.07, 3.93)	2.37 (−0.63, 5.23)	F

The above data represent the confidence interval. The bold font indicates that there was a statistically significant difference between the two treatments. A, DMARDs, B, Moxibustion + DMARDs, C, Acupuncture + DMARDs, D, Electro-acupuncture + DMARDs, E, Warm needle + DMARDs, F, Fire needle + DMARDs.

**Table 7:** Probability ranking results of VAS scores.

Drug	Rank 1	Rank 2	Rank 3	Rank 4	Rank 5	Rank 6
A	**0.9**	0.09	0.01	0	0	0
B	0.01	0.49	**0.36**	0.1	0.03	0
C	0.01	0.09	0.25	**0.47**	0.17	0
D	0	0.03	0.08	0.22	**0.59**	0.09
E	0.08	**0.29**	0.29	0.18	0.12	0.04
F	0	0	0.01	0.02	0.09	**0.87**

The bold font represents the probability ranking of the therapies. A, DMARDs, B, Moxibustion + DMARDs, C, Acupuncture + DMARDs, D, Electro-acupuncture + DMARDs, E, Warm needle + DMARDs, F, Fire needle + DMARDs.

#### 3.5.4 Results of Morning Stiffness Time

Fourteen studies reported morning stiffness time (21–24, 26–28, 31, 37, 42, 43, 46, 49, 52). The results of convergence evaluation showed that the PSRF value was close to 1, and the inconsistent fit model result was similar to the consistent fit model (*p* = 0.843 > 0.05), indicating fine stability and consistency of results, so the MCMC fitting consistency model was used for analysis. The results showed that there was no statistically significant difference between the therapies (**Table 8**), indicating that the combined therapies were not better than DMARDs in improving morning stiffness time. The probability ranking results are shown in **Table 9**.

**Table 8:** NMA results of morning stiffness time.

A	−7.62 (−20.65, 5.64)	−6.58 (−24.14, 10.95)	−16.66 (−47.36, 14.65)	−0.48 (−24.50, 23.63)	−8.23 (−42.63, 25.77)
7.62 (−5.64, 20.65)	B	1.02 (−20.96, 23.29)	−9.13 (−42.29, 24.00)	7.04 (−20.66, 34.46)	−0.69 (−36.75, 36.38)
6.58 (−10.95, 24.14)	−1.02 (−23.29, 20.96)	C	−10.19 (−41.37, 20.89)	5.90 (−23.51, 36.17)	−1.88 (−39.63, 36.95)
16.66 (−14.65, 47.36)	9.13 (−24.00, 42.29)	10.19 (−20.89, 41.37)	D	16.23 (−22.61, 55.37)	8.41 (−36.70, 53.96)
0.48 (−23.63, 24.50)	−7.04 (−34.46, 20.66)	−5.90 (−36.17, 23.51)	−16.23 (−55.37, 22.61)	E	−7.75 (−50.12, 34.57)
8.23 (−25.77, 42.63)	0.69 (−36.38, 36.75)	1.88 (−36.95, 39.63)	−8.41 (−53.96, 36.70)	7.75 (−34.57, 50.12)	F

The above data represent the confidence interval. The bold font indicates that there was a statistically significant difference between the two treatments. A, DMARDs, B, Moxibustion + DMARDs, C, Acupuncture + DMARDs, D, Electro-acupuncture + DMARDs, E, Warm needle + DMARDs, F, Auricular Needle + DMARDs.

**Table 9:** Probability ranking results of morning stiffness time.

Drug	Rank 1	Rank 2	Rank 3	Rank 4	Rank 5	Rank 6
A	0.24	0.41	0.26	0.08	0.01	0
B	0.04	0.11	0.21	0.29	0.25	0.1
C	0.09	0.14	0.2	0.26	0.22	0.08
D	0.06	0.05	0.07	0.11	0.2	0.5
E	0.36	0.18	0.15	0.13	0.11	0.06
F	0.21	0.1	0.11	0.13	0.2	0.25

A, DMARDs, B, Moxibustion + DMARDs, C, Acupuncture + DMARDs, D, Electro-acupuncture + DMARDs, E, Warm needle + DMARDs, F, Auricular Needle + DMARDs.

#### 3.5.5 Results of CRP

Twenty-seven studies reported CRP (21–24, 27–33, 35, 37, 39–52). The results of convergence evaluation showed that the PSRF value was close to 1, and the inconsistent fit model result was similar to the consistent fit model (*p* = 0.052 > 0.05), indicating fine stability and consistency of results, so the MCMC fitting consistency model was used for analysis. The results showed that Moxibustion + DMARDs was better than DMARDs, Acupuncture + DMARDs was better than DMARDs, Electro-acupuncture + DMARDs was better than DMARDs, Warm needle + DMARDs was better than DMARDs, and Fire needle + DMARDs was better than DMARDs, Moxibustion + DMARDs, Acupuncture + DMARDs, Electro-acupuncture + DMARDs, Warm needle + DMARDs, and Auricular needle + DMARDs. There was no statistically significant difference between the other different therapies, as seen in **Table 10**. The probability ranking results of reducing CRP were as follows: Fire needle + DMARDs > Electro-acupuncture + DMARDs > Acupuncture + DMARDs > Moxibustion + DMARDs > Warm needle + DMARDs > Auricular Needle + DMARDs > DMARDs, as shown in **Table 11**.

**Table 10:** NMA results of CRP.

A	−3.84 (−6.08, −1.59)	−4.97 (−6.91, −2.98)	−6.86 (−9.46, −4.17)	−3.62 (−6.88, −0.49)	−3.49 (−8.30, 1.29)	−11.90 (−16.06, −7.76)
**3.84 (1.59, 6.08)**	B	−1.14 (−4.11, 1.89)	−3.07 (−6.31, 0.41)	0.21 (−3.72, 4.07)	0.33 (−4.92, 5.66)	−8.06 (−12.79, −3.32)
**4.97 (2.98, 6.91)**	1.14 (−1.89, 4.11)	C	−1.92 (−4.95, 1.24)	1.36 (−2.57, 5.02)	1.48 (−3.82, 6.73)	−6.92 (−10.71, −3.27)
**6.86 (4.17, 9.46)**	3.07 (−0.41, 6.31)	1.92 (−1.24, 4.95)	D	3.26 (−0.98, 7.34)	3.39 (−2.28, 8.83)	−5.01 (−9.89, −0.27)
**3.62 (0.49, 6.88)**	−0.21 (−4.07, 3.72)	−1.36 (−5.02, 2.57)	−3.26 (−7.34, 0.98)	E	0.12 (−5.65, 5.93)	−8.30 (−13.59, −2.97)
3.49 (−1.29, 8.30)	−0.33 (−5.66, 4.92)	−1.48 (−6.73, 3.82)	−3.39 (−8.83, 2.28)	−0.12 (−5.93, 5.65)	F	−8.40 (−14.77, −1.93)
**11.90 (7.76, 16.06)**	**8.06 (3.32, 12.79)**	**6.92 (3.27, 10.71)**	**5.01 (0.27, 9.89)**	**8.30 (2.97, 13.59)**	**8.40 (1.93, 14.77)**	G

The above data represent the confidence interval. The bold font indicates that there was a statistically significant difference between the two treatments. A, DMARDs, B, Moxibustion + DMARDs, C, Acupuncture + DMARDs, D, Electro-acupuncture + DMARDs, E, Warm needle + DMARDs, F, Auricular Needle + DMARDs, G, Fire needle + DMARDs.

**Table 11:** Probability ranking results of CRP.

Drug	Rank 1	Rank 2	Rank 3	Rank 4	Rank 5	Rank 6	Rank 7
A	**0.92**	0.08	0.00	0.00	0.00	0.00	0.00
B	0.00	0.23	0.35	**0.29**	0.12	0.02	0.00
C	0.00	0.04	0.14	0.30	**0.46**	0.07	0.00
D	0.00	0.01	0.01	0.05	0.15	**0.77**	0.02
E	0.01	0.31	**0.30**	0.20	0.13	0.04	0.00
F	0.07	**0.34**	0.20	0.16	0.14	0.08	0.01
G	0.00	0.00	0.00	0.00	0.00	0.02	**0.97**

The bold font represents the probability ranking results of the therapy. A, DMARDs, B, Moxibustion + DMARDs, C, Acupuncture + DMARDs, D, Electro-acupuncture + DMARDs, E, Warm needle + DMARDs, F, Auricular Needle + DMARDs, G, Fire needle + DMARDs.

#### 3.5.6 Results of ESR

Twenty-six studies reported ESR (21–24, 27–33, 35, 37, 39–43, 45–52). The results of convergence evaluation showed that the PSRF value was close to 1, and the inconsistent fit model result was similar to the consistent fit model (*p* = 0.768 > 0.05), indicating fine stability and consistency of results, so the MCMC fitting consistency model was used for analysis. The results showed that Moxibustion + DMARDs was better than DMARDs, Acupuncture + DMARDs was better than DMARDs, Electro-acupuncture + DMARDs was better than DMARDs, Warm needle + DMARDs was better than DMARDs, Auricular Needle + DMARDs was better than DMARDs, and Fire needle + DMARDs was better than DMARDs, Acupuncture + DMARDs, Electro-acupuncture + DMARDs, and Warm needle + DMARDs. There was no statistically significant difference between the other treatments, as seen in **Table 12**. The probability ranking results of reducing ESR were as follows: Fire needle + DMARDs > Auricular Needle + DMARDs > Electro-acupuncture + DMARDs > Moxibustion + DMARDs > Acupuncture + DMARDs > Warm needle + DMARDs > DMARDs, as shown in **Table 13**.

**Table 12:** NMA results of ESR.

A	−11.03 (−14.79, −7.35)	−8.49 (−11.41, −5.55)	−11.11 (−14.48, −7.68)	−8.44 (−13.64, −3.13)	−11.08 (−17.82, −4.50)	−17.82 (−23.83, −12.10)
**11.03 (7.35, 14.79)**	B	2.57 (−2.13, 7.33)	−0.07 (−5.09, 4.99)	2.56 (−3.95, 9.18)	−0.05 (−7.72, 7.61)	−6.84 (−13.63, 0.02)
**8.49 (5.55, 11.41)**	−2.57 (−7.33, 2.13)	C	−2.64 (−6.81, 1.46)	0.03 (−5.96, 6.08)	−2.60 (−9.93, 4.63)	−9.34 (−14.51, −4.46)
**11.11 (7.68, 14.48)**	0.07 (−4.99, 5.09)	2.64 (−1.46, 6.81)	D	2.65 (−3.60, 9.01)	0.05 (−7.53, 7.43)	−6.70 (−13.32, −0.18)
**8.44 (3.13, 13.64)**	−2.56 (−9.18, 3.95)	−0.03 (−6.08, 5.96)	−2.65 (−9.01, 3.60)	E	−2.61 (−11.32, 5.75)	−9.39 (−17.36, −1.91)
**11.08 (4.50, 17.82)**	0.05 (−7.61, 7.72)	2.60 (−4.63, 9.93)	−0.05 (−7.43, 7.53)	2.61 (−5.75, 11.32)	F	−6.80 (−15.65, 1.98)
**17.82 (12.10, 23.83)**	6.84 (−0.02, 13.63)	**9.34 (4.46, 14.51)**	**6.70 (0.18, 13.32)**	**9.39 (1.91, 17.36)**	6.80 (−1.98, 15.65)	G

The above data represent the confidence interval. The bold font indicates that there was a statistically significant difference between the two treatments. A, DMARDs, B, Moxibustion + DMARDs, C, Acupuncture + DMARDs, D, Electro-acupuncture + DMARDs, E, Warm needle + DMARDs, F, Auricular Needle + DMARDs, G, Fire needle + DMARDs.

**Table 13:** Probability ranking results of ESR.

Drug	Rank 1	Rank 2	Rank 3	Rank 4	Rank 5	Rank 6	Rank 7
A	**1.00**	0.00	0.00	0.00	0.00	0.00	0.00
B	0.00	0.05	0.13	**0.24**	0.30	0.27	0.01
C	0.00	0.36	**0.40**	0.17	0.06	0.01	0.00
D	0.00	0.03	0.10	0.25	**0.34**	0.27	0.01
E	0.00	**0.42**	0.24	0.16	0.10	0.07	0.00
F	0.00	0.14	0.13	0.17	0.19	**0.32**	0.05
G	0.00	0.00	0.00	0.00	0.02	0.06	**0.92**

The bold font represents the probability ranking of the therapy. A, DMARDs, B, Moxibustion + DMARDs, C, Acupuncture + DMARDs, D, Electro-acupuncture + DMARDs, E, Warm needle + DMARDs, F, Auricular Needle + DMARDs, G, Fire needle + DMARDs.

#### 3.5.7 Results of RF

Twenty-three studies reported RF (21–23, 26, 28–30, 32, 33, 35–37, 40–42, 44–50, 52). Those were all indirect comparisons to form a closed loop, and consistency test was not performed. The convergence evaluation results showed that the PSRF value was close to 1, indicating stable results. Therefore, the MCMC fitting consistency model was used for analysis. The results showed that Moxibustion + DMARDs was better than DMARDs, Acupuncture + DMARDs, and Warm needle + DMARDs. There was no statistically significant difference between the other different therapies, as seen in **Table 14**. The probability ranking results of improving ESR were as follows: Moxibustion + DMARDs > Fire needle + DMARDs > Warm needle + DMARDs > Acupuncture + DMARDs > DMARDs > Electro-acupuncture + DMARDs, as shown in **Table 15**.

**Table 14:** NMA results of RF.

A	−49.15 (−92.94, −22.79)	−10.83 (−41.53, 15.19)	−3.16 (−58.27, 44.23)	−14.86 (−44.23, 14.70)	−24.36 (−78.99, 23.42)
**49.15 (22.79, 92.94)**	B	36.73 (4.69, 92.00)	43.50 (−3.68, 115.86)	32.97 (0.85, 91.15)	21.97 (−24.18, 96.10)
10.83 (−15.19, 41.53)	**−36.73 (−92.00, −4.69)**	C	7.32 (−53.45, 64.63)	−4.22 (−41.62, 38.23)	−13.66 (−55.83, 27.82)
3.16 (−44.23, 58.27)	−43.50 (−115.86, 3.68)	−7.32 (−64.63, 53.45)	D	−11.91 (−66.20, 52.95)	−21.47 (−90.03, 53.87)
14.86 (−14.70, 44.23)	**−32.97 (−91.15, −0.85)**	4.22 (−38.23, 41.62)	11.91 (−52.95, 66.20)	E	−9.44 (−71.79, 45.46)
24.36 (−23.42, 78.99)	−21.97 (−96.10, 24.18)	13.66 (−27.82, 55.83)	21.47 (−53.87, 90.03)	9.44 (−45.46, 71.79)	F

The above data represent the confidence interval. The bold font indicates that there was a statistically significant difference between the two treatments. A, DMARDs, B, Moxibustion + DMARDs, C, Acupuncture + DMARDs, D, Electro-acupuncture + DMARDs, E, Warm needle + DMARDs, F, Fire needle + DMARDs.

**Table 15:** Probability ranking results of improving RF.

Drug	Rank 1	Rank 2	Rank 3	Rank 4	Rank 5	Rank 6
A	**0.41**	**0.43**	0.12	0.03	0.01	0
B	0	0	0	0.02	0.15	**0.82**
C	0.07	0.19	**0.39**	0.29	0.06	0
D	0.39	0.19	0.15	0.13	0.11	0.03
E	0.06	0.13	0.25	**0.33**	0.22	0.01
F	0.06	0.06	0.09	0.19	**0.45**	0.14

The bold font represents the probability ranking of the therapy. A, DMARDs, B, Moxibustion + DMARDs, C, Acupuncture + DMARDs, D, Electro-acupuncture + DMARDs, E, Warm needle + DMARDs, F, Fire needle + DMARDs.

#### 3.5.8 Small Sample Effect Estimation

**Figure 9:**
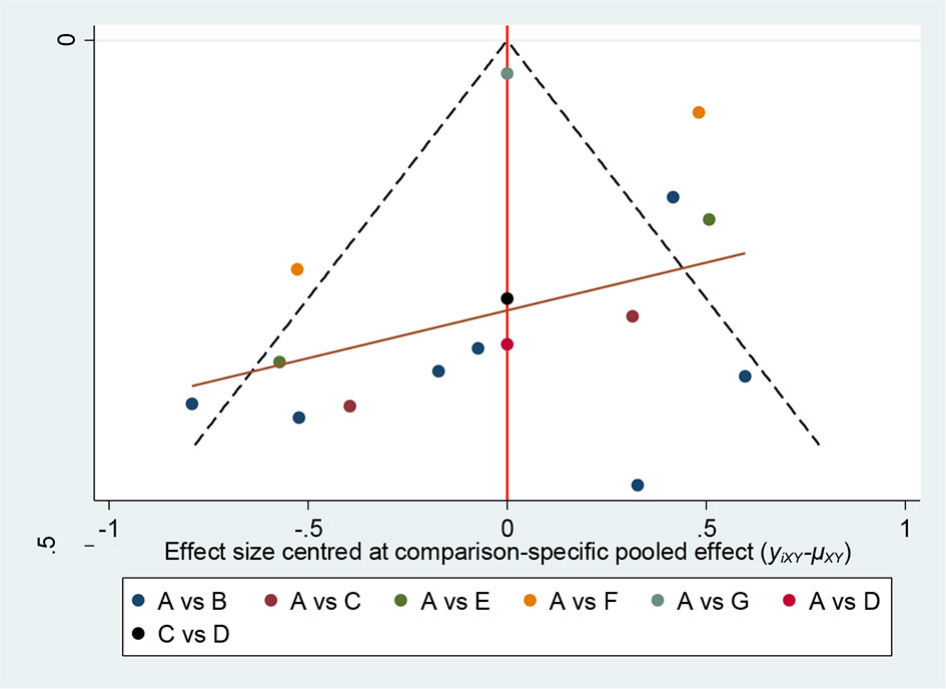
Comparison-correction funnel plot of DAS28 score. A, DMARDs, B, Moxibustion + DMARDs, C, Acupuncture + DMARDs, D, Electro-acupuncture + DMARDs, E, Warm needle + DMARDs, F, Acupoint catgut embedding + DMARDs, G, Auricular needle + DMARDs.

Comparison-correction funnel plot of the main outcome indicator, DAS28 score, was drawn by Stata 14.2 software for evaluation, as shown in **Figure 9**. The results showed that the funnel plot was not completely symmetrical, suggesting that there might be a certain publication bias or small sample effect in the research network.

### 3.6 Adverse Reactions

Ten studies reported adverse reactions (23, 28, 30, 32, 34, 35, 39, 43, 50, 51), as shown in **Table 16**. On the whole, the number of adverse reactions of different acupuncture therapies combined with DMARDs was lower than that of DMARDs, and there are no serious adverse reactions reported.

**Table 16:** Adverse reaction of the included studies.

Study	Reported adverse reactions
Wang Y 2021 (23)	Moxibustion + DMARDs: one case (vesicle). DMARDs: none.
Jiang L 2020 (28)	Acupuncture + DMARDs: none. DMARDs: two cases (oral ulceration and stomach discomfort, nausea)
Li Y 2010 (30)	Acupuncture + DMARDs: none. DMARDs: none.
Lu YL 2016 (32)	Warm needle + DMARDs: five cases (mild abdominal distension and discomfort). DMARDs: four cases (mild abdominal distension and discomfort).
Ma ZY 2016 (34)	Acupoint catgut embedding + DMARDs: three cases (slightly reddish and swollen skin). DMARDs: seven cases (2 cases of toxic adverse reactions and 5 cases of digestive tract discomfort).
Mu Y 2020 (35)	Fire needle + DMARDs: none. Acupuncture + DMARDs: one case (nausea).
Wang SQ 2018 (39)	Acupuncture + DMARDs: two cases (diarrhea and fatigue). DMARDs: 10 cases (loss of appetite, diarrhea, fatigue, fever, black stools).
Wu Y 2016 (43)	Warm needle + DMARDs: eight cases (lip ulcer, nausea, vomiting, gastritis, and diarrhea). DMARDs: six cases (lip ulcer, nausea, vomiting, gastritis, and diarrhea).
Zhang YD 2019 (50)	Fire needle + DMARDs: two cases (acid reflux and decreased appetite). Acupuncture + DMARDs: three cases (dizzy, vomit, and decreased appetite).
Zheng HY 2017 (51)	Electro-acupuncture + DMARDs: none. DMARDs: three cases (2 cases excluded and 1 case with seriously impaired liver function).

## 4 Discussion

At present, the pathogenesis of RA is not fully understood, and it might be related to autoimmunity, infection, and heredity (54). Regardless of the length of the disease, early effective treatment of RA could reduce the disability, control disease activity, and prevent and delay the patient’s condition (55, 56). Studies have shown that the use of non-steroidal anti-inflammatory drugs, DMARDs, and steroid would cause serious side effects, and drug resistance in some patients, which may seriously reduce the therapeutic effect (57). Acupuncture, as a reliable and safe alternative therapy, plays an important role in the treatment of RA (58). The efficacy and safety of acupuncture combined with DMARDs in the treatment of RA have been clinically verified, but the selection of the optimal combination has become a current research priority.

In this study, we evaluated the effects of acupuncture-related therapies combined with DMARDs on DAS28, VAS, morning stiffness time, CRP, ESR, and RF in patients with RA. DAS28 could continuously measure RA disease activity with information of swollen joints, tender joints, acute phase response, and general health, and it has been widely used to evaluate the remission of RA patients (59). Morning stiffness and pain are the main symptoms that accompany the progression of RA, which can reflect the severity of RA. Generally speaking, the longer the morning stiffness and the more severe the pain was, the worse the condition was (60). Serological disease markers (CRP, ERS, and RF) are important indicators for judging the active stage of RA, which can reflect the degree of inflammation and tissue damage in patients (61). In particular, RF is an important indicator for diagnosing RA and judging its prognosis (62). The results of our study showed that in terms of improving DAS28 scores, electro-acupuncture combined with DMARDs had the best effect according to the probability ranking results, and Electro-acupuncture + DMARDs was superior to Moxibustion + DMARDs, DMARDs, and Acupoint catgut embedding + DMARDs according to the NMA results. In terms of improving VAS score, fire needle combined with DMARDs had the best effect according to the probability ranking results, and the NMA results showed that Fire needle + DMARDs was better than Moxibustion + DMARDs, Acupuncture + DMARDs, and DMARDs. In terms of improving morning stiffness time, there was no statistically significant difference between all the therapies, which meant that acupuncture-related therapies combined with DMARDs were not better than the use of DMARDs alone in improving morning stiffness in RA patients. In terms of reducing CRP, fire needle combined with DMARDs had the best effect according to the probability ranking results, while the NMA results showed that Fire needle + DMARDs was better than DMARDs, Moxibustion + DMARDs, Acupuncture + DMARDs, Electro-acupuncture + DMARDs, Warm needle +DMARDs, and Auricular needle + DMARDs. In terms of reducing ESR, the probability ranking results showed that fire needle combined with DMARDs had the best effect, and the NMA results showed that Fire needle + DMARDs was better than DMARDs, Acupuncture + DMARDs, Electro-acupuncture + DMARDs, and Warm needle + DMARDs. In terms of reducing RF, the probability ranking results showed that Moxibustion + DMARDs had the best effect, followed by Fire needle + DMARDs, while the NMA results showed that Moxibustion + DMARDs was superior to DMARDs, Acupuncture + DMARDs, and Warm needle + DMARDs, but there was no significant difference between Moxibustion + DMARDs and Fire needle + DMARDs. The above results indicated that though the curative effects of different indicators were different, Electro-acupuncture + DMARDs, Fire needle + DMARDs, and Moxibustion + DMARDs ranked first among the multiple indicators. It can be seen that Electro-acupuncture + DMARDs, Fire needle + DMARDs, and Moxibustion + DMARDs have outstanding efficacy in the treatment of RA. Considering that the quality of the included studies is moderate, it is necessary to make a reasonable selection based on the characteristics of the patient’s condition in clinical practice.

According to the results of the study, the top rankings are as follows: Fire needle combined with DMARDs, Electro-acupuncture combined with DMARD, and Moxibustion combined with DMARD. Modern studies have found that acupuncture can effectively relieve the pain and improve the quality of life in RA patients. The curative effect is related to anti-inflammation, antioxidant, immune system, endorphins, and serotonin (13, 63). Fire needle, electro-acupuncture and moxibustion are further improved and developed on the basis of acupuncture theory, which could enhance the curative effect. Fire needle could effectively inhibit inflammation of RA by downregulating Anti-cyclic citrullinated peptide antibody (ACPA) and tumor necrosis factor-α (TNF-a) (64). Electro-acupuncture can reduce the levels of TNF-α and vascular endothelial growth factor (VEGF) in peripheral blood and synovium of joints, improve the internal environment, and relieve joint symptoms of RA patients (65). Moxibustion could downregulate the levels of interleukin-1β (IL-1β), TNF-α, matrix metalloproteinase 1 (MMP-1), matrix metalloproteinase 3 (MMP-3), and hypoxia-inducible factor-1α (HIF-1α)/VEGF, and inhibit angiogenesis to show a potential protective effect on bones (66). For the commonly used acupoints, modern studies have shown that stimulation of ST36 with electro-acupuncture could activate the anti-inflammatory pathway of vagus nerve-adrenal gland in mice to exert anti-inflammatory effects (67), while stimulating Ashi points can inhibit the expression of phosphorylated c-Jun N-terminal kinase in dorsal root ganglion of mice and thus play an analgesic role (68).

There are many limitations in this study. First, many studies included did not specifically report random methods, allocation concealment, and blinding, which influenced the testing power of the research results. Second, the sample size of the included studies was small, which might limit the accuracy of the results. Third, the type and dosage of DMARDs, the point selection of acupuncture-related therapies, and the course of treatment were different in the included studies, which might increase clinical heterogeneity. Fourth, there was certain publication bias and small sample effects in the studies, which might influence the reliability of the results. Fifth, there is a lack of other acupuncture therapies, such as bloodletting therapy and acupoint injection, because of the limited amount of original studies, which made it impossible to compare the efficacy of all acupuncture-related therapies. Sixth, as for DAS28 score, it is not clear whether DMARDs combined with fire acupuncture is superior to Electro-acupuncture combined with DMARDs because the treatment of DMARDs combined with fire acupuncture was not included in the primary outcome indicators.

In conclusion, after a comprehensive comparison of the outcome indicators of 8 different therapies, electro-acupuncture combined with DMARDs is the best therapy to improve the DAS28 score. In terms of improving pain and serological markers, fire needle combined with DMARDs and moxibustion combined with DMARDs are the best, but it is impossible to tell which is better. In clinical practice, the appropriate treatment method should be selected according to the actual situation. Due to the current limited literature reports and the poor quality of some of them, more multi-center, large-sample, prospective RCT studies are needed to verify the conclusions.

## Data Availability Statement

The original contributions presented in the study are included in the article/**Supplementary Material**. Further inquiries can be directed to the corresponding authors.

## Author Contributions

This study was conceived by RW and YF. RW YF and AZ drafted the manuscript. RW, YF, YX, and XH participated in the design of the data synthesis analysis scheme. LZ and YW provided oversight, critical evaluation, and verification of the manuscript. All authors contributed to the article and approved the submitted version.

## Funding

This work was supported by the Taizhou Science and Technology Project (No.21ywb70). Jiangxi Provincial Department of Science and Technology Key RESEARCH and Development Program General Project (No. 20192BBGL70037).

## Conflict of Interest

The authors declare that the research was conducted in the absence of any commercial or financial relationships that could be construed as a potential conflict of interest.

## Publisher’s Note

All claims expressed in this article are solely those of the authors and do not necessarily represent those of their affiliated organizations, or those of the publisher, the editors and the reviewers. Any product that may be evaluated in this article, or claim that may be made by its manufacturer, is not guaranteed or endorsed by the publisher.

## Supplementary Material

Supplementary Table S1 The PRISMA checklist.
