# Supplementary material for: Comparison of Efficacy of Acupuncture-Related Therapy in the Treatment of Rheumatoid Arthritis: A Network Meta-Analysis of Randomized Controlled Trials
Source: Front Immunol. 2022 Mar 7;13:829409. doi: 10.3389/fimmu.2022.829409 (PMC8936080; doi:10.3389/fimmu.2022.829409)
Supplement: Supplementary file 2 [file Table_2.docx]

| **Table S2. Direct comparison of meta-analysis results** | | | | | | |
| --- | --- | --- | --- | --- | --- | --- |
| Outcome index | Comparison category | Number of studies | Heterogeneity | | Meta analysis results | |
|  |  |  | *I^2^* | *P* | MD/SMD, 95%CI | *P* |
|  | Moxibustion + DMARDs vs DMARDs | 6 | 69% | 0.006 | **-1.01 (-1.63, -0.40)** | **0.001** |
|  | Acupuncture + DMARDs vs DMARDs | 3 | 73% | 0.03 | **-1.56( -2.94, -0.19)** | **0.03** |
|  | Eletro-acupuncture + DMARDs vs DMARDs | 2 | 8% | 0.3 | **-2.19(-2.77, -1.61)** | **<0.00001** |
|  | Warm needle + DMARDs vs DMARDs | 1 | NA | NA | **-1.23( -1.92, -0.54)** | **0.0005** |
|  | Fire needle + DMARDs vs DMARDs | 1 | NA | NA | **-1.95( -2.38, -1.52)** | **<0.00001** |
| Disease Activity Score of 28 Joints (DAS28) scores |  |  |  |  |  |  |
|  | Moxibustion + DMARDs vs DMARDs | 7 | 64% | 0.01 | **-0.62( -1.00, -0.24)** | **0.001** |
|  | Acupuncture + DMARDs vs DMARDs | 2 | 59% | 0.12 | **-0.92(-1.61, -0.22)** | **0.009** |
|  | Eletro-acupuncture + DMARDs vs DMARDs | 1 | NA | NA | **-2.00(-2.59, -1.41)** | **<0.00001** |
|  | Eletro-acupuncture + DMARDs vs Acupuncture + DMARDs | 1 | NA | NA | **-1.40(-1.90, -0.90)** | **<0.00001** |
|  | Warm needle + DMARDs vs DMARDs | 2 | 89% | 0.003 | -0.83(-1.88,0.23) | 0.12 |
|  | Acupoint catgut embedding + DMARDs vs DMARDs | 2 | 94% | <0.0001 | 0.28( -0.71, 1.27) | 0.58 |
|  | Auricular needle + DMARDs vs DMARDs | 1 | NA | NA | **-1.24(-1.30, -1.18)** | **<0.00001** |
| Morning stiffness time |  |  |  |  |  |  |
|  | Moxibustion + DMARDs vs DMARDs | 7 | 89% | <0.00001 | -0.58(-1.28, 0.11) | 0.10 |
|  | Acupuncture + DMARDs vs DMARDs | 4 | 0% | 0.84 | **-0.85(-1.12, -0.59)** | **<0.00001** |
|  | Eletro-acupuncture + DMARDs vs DMARDs | 1 | NA | NA | **-2.07(-2.85, -1.29)** | **<0.00001** |
|  | Eletro-acupuncture + DMARDs vs Acupuncture + DMARDs | 1 | NA | NA | **-1.14(-1.82, -0.47)** | **0.0009** |
|  | Warm needle + DMARDs vs DMARDs | 2 | 40% | 0.20 | **-0.59(-0.88, -0.29)** | **0.0001** |
|  | Auricular needle + DMARDs vs DMARDs | 1 | NA | NA | **-2.67(-3.37, -1.96)** | **<0.00001** |
| C-reactive protein  (CRP) |  |  |  |  |  |  |
|  | Moxibustion + DMARDs vs DMARDs | 11 | 46% | 0.05 | **-4.19(-5.49, -2.89)** | **<0.00001** |
|  | Acupuncture + DMARDs vs DMARDs | 7 | 72% | 0.001 | **-5.44(-6.88, -4.00)** | **<0.00001** |
|  | Eletro-acupuncture + DMARDs vs DMARDs | 4 | 93% | <0.00001 | **-6.68(-10.91, -2.44)** | **0.002** |
|  | Eletro-acupuncture + DMARDs vs Acupuncture + DMARDs | 1 | NA | NA | **-4.00(-5.57, -2.43)** | **<0.00001** |
|  | Warm needle + DMARDs vs DMARDs | 3 | 30% | 0.24 | **-3.40(-4.42, -2.38)** | **<0.00001** |
|  | Auricular needle + DMARDs vs DMARDs | 1 | NA | NA | **-3.49(-3.77, -3.21)** | **<0.00001** |
|  | Fire needle + DMARDs vs Acupuncture + DMARDs | 2 | 70% | 0.07 | **-6.75(-9.20, -4.29)** | **<0.00001** |
| Erythrocyte Sedimentation Rate  (ESR) |  |  |  |  |  |  |
|  | Moxibustion + DMARDs vs DMARDs | 10 | 38% | 0.11 | **-10.47(-13.22, -7.73)** | **<0.00001** |
|  | Acupuncture + DMARDs vs DMARDs | 7 | 82% | <0.00001 | **-8.65(-11.28, -6.01)** | **<0.00001** |
|  | Eletro-acupuncture + DMARDs vs DMARDs | 4 | 64% | 0.04 | **-10.59(-12.98, -8.20)** | **<0.00001** |
|  | Eletro-acupuncture + DMARDs vs Acupuncture + DMARDs | 1 | NA | NA | **-5.00(-7.51, -2.49)** | **<0.0001** |
|  | Warm needle + DMARDs vs DMARDs | 3 | 0% | 0.67 | **-8.81(-11.35, -6.67)** | **<0.00001** |
|  | Auricular needle + DMARDs vs DMARDs | 1 | NA | NA | **-11.02(-12.10, -9.94)** | **<0.00001** |
|  | Fire needle + DMARDs vs Acupuncture + DMARDs | 2 | 87% | 0.006 | **-9.52(-15.03, -4.01)** | **0.0007** |
| Rheumatoid Factor (RF) |  |  |  |  |  |  |
|  | Moxibustion + DMARDs vs DMARDs | 10 | 65% | 0.002 | **-0.50 (-0.80, -0.20)** | **0.001** |
|  | Acupuncture + DMARDs vs DMARDs | 5 | 90% | <0.00001 | **-1.19(-1.93, -0.45)** | **0.002** |
|  | Eletro-acupuncture + DMARDs vs DMARDs | 2 | 72% | 0.06 | **-0.62(-1.22, -0.02)** | **0.04** |
|  | Warm needle + DMARDs vs DMARDs | 4 | 56% | 0.08 | **-0.86(-1.20, -0.52)** | **<0.00001** |
|  | Fire needle + DMARDs vs Acupuncture + DMARDs | 2 | 97% | <0.00001 | -2.64(-5.40, 0.12) | 0.06 |
| Abbreviation：NA, Data unavailable; MD, Mean Difference; SMD, Standard Mean Difference; CI, Confidence interval. The bold font indicates that there was a statistically significant difference between the two treatments. | | | | | | |
