# Supplementary material for: Comparison of Efficacy of Acupuncture-Related Therapy in the Treatment of Rheumatoid Arthritis: A Network Meta-Analysis of Randomized Controlled Trials
Source: Front Immunol. 2022 Mar 7;13:829409. doi: 10.3389/fimmu.2022.829409 (PMC8936080; doi:10.3389/fimmu.2022.829409)
Supplement: Supplementary file 3 [file Table_3.docx]

**Table S3. Results of subgroup analysis**

| Outcome index | Treatment course (week) | Number of studies | Heterogeneity | | Meta analysis results | |
| --- | --- | --- | --- | --- | --- | --- |
|  |  |  | *I^2^* | *P* | MD/SMD, 95%CI | *P* |
| Disease Activity Score of 28 Joints (DAS28) scores | 8 | 3 | 0% | 0.63 |  |  |
|  |  |  |  |  | **-0.84 (-1.22, -0.47)** | **<0.00001** |
|  | 12 | 4 | 72% | 0.01 | **-0.45( -1.02, -0.11)** | **0.001** |
| Visual Analogue Scale (VAS) scores |  |  |  |  |  |  |
|  | 8 | 3 | 0% | 0.38 | **-0.93 (-1.40, -0.45)** | **0.0001** |
|  | 12 | 3 | 86% | 0.10 | -1.10(-2.42, -0.22) | 0.10 |
| Morning stiffness time |  |  |  |  |  |  |
|  | 8 | 3 | 0% | 0.76 | -0.60 (-0.89, -0.31) | **<0.0001** |
|  | 12 | 4 | 94% | <0.00001 | -0.66(-2.16, 0.84) | 0.39 |
| C-reactive protein  (CRP) |  |  |  |  |  |  |
|  | ≥4 and ≤8 | 6 | 63% | 0.02 | **-3.45(-5.83, -1.06)** | **0.005** |
|  | 12 | 5 | 14% | 0.32 | **-5.86(-11.27, -0.46)** | **0.03** |
| Erythrocyte Sedimentation Rate  (ESR) |  |  |  |  |  |  |
|  | ≥4 and ≤8 | 5 | 36% | 0.18 | **-8.57(-11.95, -5.20)** | **<0.00001** |
|  | 12 | 5 | 15% | 0.32 | **-14.18(-18.89, -9.47)** | **<0.00001** |
| Rheumatoid Factor  (RF) |  |  |  |  |  |  |
|  | ≥4 and ≤8 | 5 | 4% | 0.38 | **-0.26(-0.47, -0.04)** | **0.02** |
|  | 12 | 5 | 74% | 0.004 | **-0.83(-1.45, -0.21)** | **0.008** |

Abbreviation：MD, Mean Difference; SMD, Standard Mean Difference; CI, Confidence interval. The bold font indicates that there was a statistically significant difference between the two treatments.
